# A Decade of GigaScience: GigaDB and the Open Data Movement

**DOI:** 10.1093/gigascience/giac053

**Published:** 2022-06-14

**Authors:** Chris Armit, Mary Ann Tuli, Christopher I Hunter

**Affiliations:** GigaScience Press, BGI Hong Kong Tech Co Ltd., 26F A Kings Wing Place 2, 1 On Kwan Street, Shek Mun, Sha Tin, NT, Hong Kong SAR; GigaScience Press, BGI Hong Kong Tech Co Ltd., 26F A Kings Wing Place 2, 1 On Kwan Street, Shek Mun, Sha Tin, NT, Hong Kong SAR; GigaScience Press, BGI Hong Kong Tech Co Ltd., 26F A Kings Wing Place 2, 1 On Kwan Street, Shek Mun, Sha Tin, NT, Hong Kong SAR

## Abstract

The increasingly multidisciplinary nature of scientific research necessitates a need for Open Data repositories that can archive data in support of publications in scientific journals. Recognising this need, even before *GigaScience* launched in 2012, GigaDB was already in place and taking data for a year before (making it 11 this year). Since GigaDB launched, there has been a consistent growth in this resource in terms of data volume, data discoverability and data re-use. In this commentary, we provide a retrospective of key changes over the last decade, and the role of Data Curation in enhancing the user experience. Furthermore we explore a much needed emphasis on enabling researchers to interact with and explore datasets prior to data download.

*“The journey of a thousand miles begins with a single step.”*

Lao Tzu

In scientific research, there is a salient need for Open Data repositories that can handle multiple data types. The multi-disciplinary nature of the omics revolution necessitates the ability to archive a diverse range of data types—including, genomic, metagenomic, transcriptomic, proteomic, and imaging data, as well as the scripts and code used in their analysis—which together provide a more holistic view of living systems. As a means of addressing this issue, the provision of a “Big Data” archiving solution has been a core tenet of GigaDB (http://gigadb.org/) since its launch, and over the last 10 years, we have seen this repository grow from 30 datasets to a rich archive of over 2100 datasets in 2022.

During this 10-year period, we have observed a major shift in the way research is shared. Data is now more open and reusable, and as a myriad number of case studies have shown there is immense value in an integrated data and research object publishing approach [1]. When *GigaScience* journal launched in 2012 it capitalised on the existence of GigaDB making it straightforward for *GigaScience* to be one of very few journals to have, and enforce, an Open Data policy. Having a strict and easily enforceable policy was one of the key goals of the journal; thus, building and launching GigaDB first was essential. Since then, and with the FAIR principles being published in 2016 [2], major scientific journals are now supporting this framework, and UNESCO recently published their Open Science Recommendations, which asks the 193 ratifying Member States to take whatever legislative or other steps required to identify and take concrete measures on Open Access and Open Data [3].

In GigaDB, there has been a significant enrichment in data discoverability over the last 10 years. Following the launch of *GigaScience*, Sneddon and colleagues [4] reported on the fledgling GigaDB repository, which by that point, only included 30 datasets (Fig. 1).

**Figure 1:**
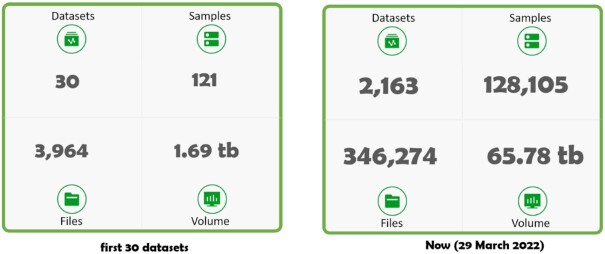
Comparison of GigaDB Dataset metrics from 2012 to 2022.

While the database had been designed with the inclusion of extensive metadata in mind, many of the early datasets had very low levels of metadata description included. This was primarily due to a general lack of awareness in some research communities on the need for rich metadata to ensure data discoverability. To help researchers improve the metadata for their work, we set up ways to improve the capture of rich metadata, primarily by the creation of a simple submission tool to guide the authors on how to add appropriate biological sample attributes. However, the key innovation for GigaDB was the creation of a dedicated Data Curation team to personally interact with authors, guiding them and asking direct questions about metadata that were tailored to each individual manuscript and the related data standards. The role of the Data Curation team is to ensure the submission process runs as smoothly as possible, resulting in expedient public release of data associated with a *GigaScience* manuscript. Data Curators liaise with authors and advise on the appropriate Ontologies and Data Standards to use for what can be quite complex datasets (genomic, software, multispectral or microCT imaging, etc) often within the same manuscript.

With the creation of a Data Curation team, we have observed a fourfold improvement in metadata over the decade (Fig. 2). As a direct consequence, extensive sample metadata, including where possible Genomics Standard Consortium (GCS)-approved sample attributes, are now provided for each new sample that is added to the GigaDB repository. Our close collaboration with the GSC in particular has further highlighted the importance of using community-driven data standards to ensure data are comprehensive and discoverable. To this end we are exploring the incorporation of other community-driven data standards, such as DOME-ML, for Artificial Intelligence/Machine Learning studies, and STORMS for the reporting of microbiomes [5]. We have experimented with pre-registration and Randomised Controlled Trials to assess if improved metadata does indeed provide measurable increases in discoverability [6]. From this study, we suggest that the curation of metadata should be understood as an ongoing process, and the Data Curation team continues to monitor and be part of the development of Data Standards.

**Figure 2:**
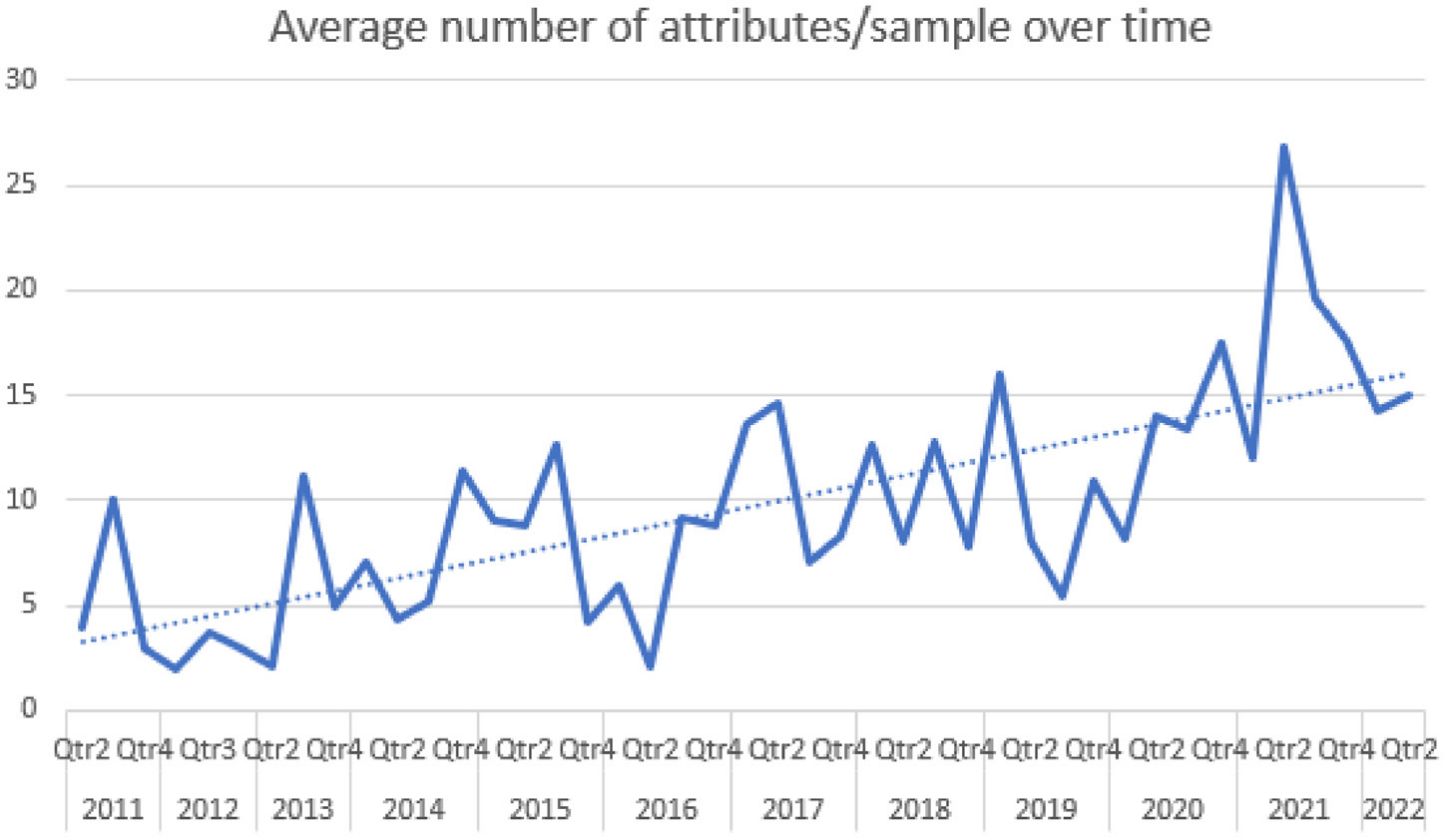
Metadata enrichment in GigaDB over the last 10 years.

Metadata adds value to datasets on multiple levels, especially by ensuring they are discoverable.

The members of the curation team have distinct but overlapping roles, which enables us to triage the datasets based on in-house expertise. In most cases, authors communicate directly with a single curator during the submission process, clarifying what data is required and advising on the best way to incorporate these data into GigaDB. Having a single point of contact enables the authors and curators to build a more personal working relationship and ensures that data is represented in GigaDB in the most appropriate way, whilst remaining consistent with other datasets. Curators communicate closely with the GigaScience Press editorial team and modify curation priorities as needed.

To further aid submitting authors, in 2019 we introduced a set of checklists for some of the more common types of manuscript submissions where we list the appropriate sample metadata and file types that are required at the pre-review and post-review stages in the *GigaScience* publication process. For GigaScience Press, this has been of immense value in ensuring a more rapid time to review, and streamlining the entire data publication process. Indeed, the rapid publication model—for which GigaDB was a critical component—ultimately led to the launch of its sister journal *GigaByte* in 2020, which aims to promote the most rapid exchange of scientific information in a formal peer-reviewed publishing platform. The checklists include a list of appropriate files one would expect to see submitted in support of, for example, a genomic or imaging study. In addition, for rapid review it is imperative that detailed file descriptions are made available to ensure a researcher can unambiguously interpret the contents of the submitted files. Furthermore, a major focus has been ensuring that file integrity is maintained through the use of MD5 checksums, which are typically requested from authors in the pre-review stage.

More recently we have started to enhance the utility of the data we host with the integration of online tools that allow researchers to directly interact with and explore datasets prior to data download [7]. We have found embeddable media to be particularly helpful in this respect, with Code Ocean (https://codeocean.com/) compute capsules as an important example, as this enables software tools associated with *GigaScience* manuscripts to be made interactively available in the context of a GigaDB dataset page [8]. These media are embedded in the GigaDB web-based platform by our in-house agile software development team. An additional example of embeddable media that is routinely used by GigaDB is SketchFab (https://sketchfab.com/), which is perfectly suited for generating interactive 3D visualisations of surface-rendered 3D microCT imaging datasets [9]. Other embeddable media currently supported by GigaDB include JBrowse [10], which is a Genome browser, Juicebox for Hi-C maps, and protocols.io [10], which is well suited for detailing methods, especially as it allows versioning and forking of methods for future papers.

## Future Work

The intention moving forward is to expand on these features to further encourage reuse of omics data. Towards this end, in 2022 we are launching a sophisticated search functionality to allow users to discover datasets of interest, and this will be accompanied with improvements to the API to facilitate computational access to data. As with all GigaDB development work, this is being accomplished using our GitHub repository (https://github.com/gigascience/gigadb-website) under an open license (GPLv3) to enable anyone to contribute to the infrastructure.

The hard work and dedication of the Data Curation team in collating structured metadata will enable appropriate filtering of search results. Furthermore, by incorporating agile practices, GigaDB is in the position of being able to quickly adapt to new and novel data types that require data sharing. With this infrastructure in place, we look forward to the continued growth of GigaDB over the next 10 years.

### Abbreviations

GigaDB: GigaScience Database; GSC: Genomics Standard Consortium

## Data Availability

Not applicable.

## Editor's Note

This commentary is part of a series to celebrate a Decade of *GigaScience*, to coincide with the 10th anniversary of *GigaScience* journal's launch in July 2012. These papers take a look back at 10 years of advances in large-scale research as open science has become mainstream.

## Competing Interests

All of the authors are employees of GigaScience Press who host and run GigaDB.
